# Seroprevalence of brucellosis in camels and humans in the Al-Qassim region of Saudi Arabia and its implications for public health

**DOI:** 10.1186/s13568-025-01822-8

**Published:** 2025-02-07

**Authors:** Abdulaziz M. Almuzaini, Abdullah S. M. Aljohani, Ahmed I. Alajaji, Ayman Elbehiry, Adil Abalkhail, Abdulrahman Almujaidel, Sahar N. Aljarallah, Hazem R. Sherif, Eman Marzouk, Abdelmaged A. Draz

**Affiliations:** 1 Department of Veterinary Preventive Medicine, College of Veterinary Medicine, Qassim University, 51452 Buraydah, Saudi Arabia; 2 Department of Medical Biosciences, College of Veterinary Medicine, Qassim University, 51452 Buraydah, Saudi Arabia; 3 Department of Public Health, College of Applied Medical Sciences, Qassim University, 51452 Buraydah, Saudi Arabia; 4 Department of Bacteriology, Mycology and Immunology, Faculty of Veterinary Medicine, University of Sadat City, Sadat City, 32511 Egypt; 5 Department of Pharmacy Sciences, College of Pharmacy, AlMaarefa University, 13713 Dariyah, Riyadh Saudi Arabia; 6 Department of Reproductive Deseases, Animal Reproductive Research Institute, Giza, Egypt; 7 Department of Animal Hygine and Zoonoses, Faculty of Veterinary Medicine, Amriya, Alexandria University, Alexandria, 21944 Egypt

**Keywords:** Brucellosis, Risk factors, Animals, Qassim, Zoonosis, Prevalence, Public health

## Abstract

Brucellosis is a significant zoonotic disease caused by intracellular, gram-negative bacteria from the genus *Brucella*. Although camels are classified as secondary hosts for *Brucella* species, they are among the most susceptible and vulnerable animals to brucellosis, particularly *Brucella abortus* (*B. abortus*) and *Brucella melitensis* (*B. melitensis*). The present study aimed to investigate the epidemiology of camel brucellosis as a zoonotic disease by determining the seroprevalence of brucellosis in both camels and humans, assessing potential risk factors (e.g., age, size, and location), and conducting molecular characterization of *Brucella* spp. associated with abortion in camels. The Rose Bengal Test (RBT), Antigen Rapid *Brucella* Antibody Test (ARBT), indirect enzyme-linked immunosorbent assay (I-ELISA), and complement fixation test (CFT) were used to detect brucellosis in both camels and humans. Additionally, a molecular method using polymerase chain reaction was used as a confirmatory technique. A total of 625 camel serum samples and 100 human serum samples were collected in sterile vacuum tubes from various camel farms and individuals across different localities in the Al Qassim region. Additionally, samples from 10 confirmed *Brucella*-infected camels (including the uterus and supramammary lymph nodes) were analyzed. The results indicated that the overall prevalence of brucellosis in camel sera was 9.72%, as determined by RBT, and 8.16%, as determined by ARBT. In contrast, the overall prevalence of brucellosis in human sera from febrile patients was found to be 17% via RBT. Notably, 57.98% of camel sera that tested positive for *Brucella* antibodies via RBT were also positive according to I-ELISA and CFT. Furthermore, 42.1%, 70.58%, and 47.05% of human sera that were positive for *Brucella* antibodies as determined by RBT were also positive according to I-ELISA and CFT, respectively. The highest seropositivity for camel brucellosis was observed in female camels, particularly in the Unaizah area of the Qassim region and among the Homr breed. The prevalence of human brucellosis was highest among females and individuals who consumed raw milk. At the molecular level, *B. melitensis* biovar 3 was detected in the examined tissues. In conclusion, intervention measures are vital for reducing brucellosis in humans and camels. Public awareness campaigns should highlight the importance of protective clothing when handling aborted she-camels and the need to boil or pasteurize milk. Additionally, studies should differentiate between vaccinated and nonvaccinated camels, and standardizing serological tests for diagnosing brucellosis should be prioritized.

**Supplementary Information:**

The online version contains supplementary material available at 10.1186/s13568-025-01822-8.

## Introduction

Brucellosis is a bacterial infection and a significant zoonotic disease of global concern that primarily affects domestic ruminants (Elbehiry et al. 2022a, b; Elbehiry et al. 2023). It is caused by intracellular, gram-negative bacteria belonging to the genus *Brucella*. The transmission of the causative bacteria to humans occurs through direct physical contact with infected animals or via the consumption of contaminated animal products (Bayasgalan et al. 2018; Franc et al. 2018; Lokamar et al. 2022). The disease is attributed to four principal etiological agents, *Brucella abortus* (*B. abortus*), *Brucella melitensis* (*B. melitensis*), *Brucella suis* (*B. suis*), and *Brucella canis* (*B. canis*), all of which pose risks to both human health and various animal populations (Głowacka et al. 2018). While goats and sheep serve as the primary hosts for *B. melitensis*, cattle are predominantly associated with *B. abortus*, and pigs are the main hosts for *B. suis*. Camels are infected by various biovars of the two species *B. abortus* and *B. melitensis*. The three species—*B. abortus*, *B. suis*, and *B. melitensis*—are recognized as the most significant *Brucella* pathogens affecting humans and are responsible for the majority of the disease burden in animals. Nevertheless, *B. canis* is also recognized as a species that may pose an infectious risk to humans (Bayasgalan et al. 2018).

*Brucella*, similar to various other intracellular pathogens, has a biphasic infection course (Deghelt et al. 2014; González-Espinoza et al. 2021). This dual-phase infection can lead to numerous complications, including infertility, abortion, decreased milk production, and significant economic losses for animal owners, as well as increased healthcare costs associated with treatment aimed at alleviating symptoms, mitigating complications, and preventing relapse of the i. Camels are among the species most susceptible to brucellosis, particularly *B. abortus* and *B. melitensis*, although they are classified as secondary hosts for *Brucella* species (Gwida et al. 2012; Elbehiry 2014; Bayasgalan et al. 2018; Elbehiry et al. 2022a, b; Mohammed et al. 2023). The economic impact on camel populations can be substantial, resulting from infertility, abortions, mastitis, and reduced milk production in affected individuals. Infertility is characterized primarily by an extended intercalving period, whereas abortion contributes to neonatal mortality in infected animals (Al-Majali 2005; Dawood et al. 2023). With over 8,000 cases reported annually, Saudi Arabia ranks among the countries with the highest prevalence of brucellosis. The Al-Qassim region, in particular, has recorded the highest incidence of brucellosis cases (Abdullah et al. 2024; Aloufi et al. 2016; Almuzaini 2023; Elbehiry et al. 2023).

The impact of brucellosis extends to consumers of products derived from infected animals, including humans. This zoonotic disease poses significant public health risks (Poester et al. 2002). According to a report by the Iowa State Center for Food Security and Public Health, brucellosis is transmitted primarily through contact with aborted fetuses, placental tissues, and other contaminated samples, as well as through the consumption of raw camel milk (Khurana et al. 2021). The necessity of preventing and controlling brucellosis prompted the development of the first serodiagnostic test, a simple tube agglutination test, which was introduced by Wright and Smith in 1897 (Weight 1897). Several additional diagnostic tests have subsequently been developed to increase testing efficiency (Deghelt et al. 2014). These tests include the milk ring test (MRT), the Rose Bengal plate test (RBT), a modified version of the serum tube agglutination test (SAT), the antiglobulin (Coombs) test, the complement fixation test (CFT), and the enzyme-linked immunosorbent assay (ELISA) (Saxena et al. 2018). The implementation of these diagnostic tests has improved the control and diagnosis of brucellosis, facilitating appropriate treatment and management when necessary.

Annually, more than half a million cases of human brucellosis are reported worldwide; however, it is important to note that a significantly greater number of undiagnosed cases exist (Al Dahouk et al. 2013; Laine et al. 2023). Over the past two centuries, the traditional epidemiology of brucellosis has undergone substantial changes, which are closely associated with various political and socioeconomic factors. For example, while a high prevalence of brucellosis persists in North African countries and the Middle East, with a few exceptions, there has been a marked decline in its incidence in Southern European and Latin American nations (Ali 2009; Al Dahouk et al. 2013).

Brucellosis is recognized as an occupational hazard that presents a significant risk of infection to laboratory personnel, abattoir workers, veterinarians, farmers, and other individuals who handle aborted fetuses or reside in proximity to infected animals, as well as those who consume contaminated animal products containing *Brucella* agents (Shoukat et al. 2017; Dadar et al. 2023). Numerous studies have indicated that raw camel milk and camel meat may serve as potential sources for the transmission of this bacterium to humans.

The rise of multidrug resistance in different bacteria poses a significant public health threat (Shafiq et al. 2022; Al-Kadmy et al. 2024; Algammal et al. 2024). Owing to its intracellular nature, treating multidrug-resistant *Brucella* is challenging, leading to complications (Elbehiry et al. 2022a, b). Brucellosis presents serious health risks with nonspecific symptoms, including depression, intermittent fever, hepatomegaly, weight loss, and splenomegaly (Mokhtar et al. 2007; Hadush et al. 2013). Additionally, some patients may experience more severe complications, such as arthritis, epididymitis, orchitis, osteomyelitis, and spondylitis, and critical conditions, such as endocarditis, liver abscesses, and neurobrucellosis (Bingöl et al. 1999; Alatabi et al. 2020; Hosein et al. 2024). Other reported symptoms in humans include arthralgia, breast abscess, fatigue, epididymal orchitis, undulant fever, headache, insomnia, joint pain, malaise, sweating, spondylitis, weight loss, and the formation of bone or testicular abscesses, as well as cardiovascular, musculoskeletal, and neurological complications (Hassan-Kadle et al. 2021). This study investigated camel brucellosis epidemiology as a zoonotic disease by determining the seroprevalence in camels and humans, assessing risk factors, and conducting molecular characterization of *Brucella* strains while analyzing the effects of sex, age, locality, season, and breed on prevalence.

## Materials and methods

### Study design and study population

**Fig. 1:**
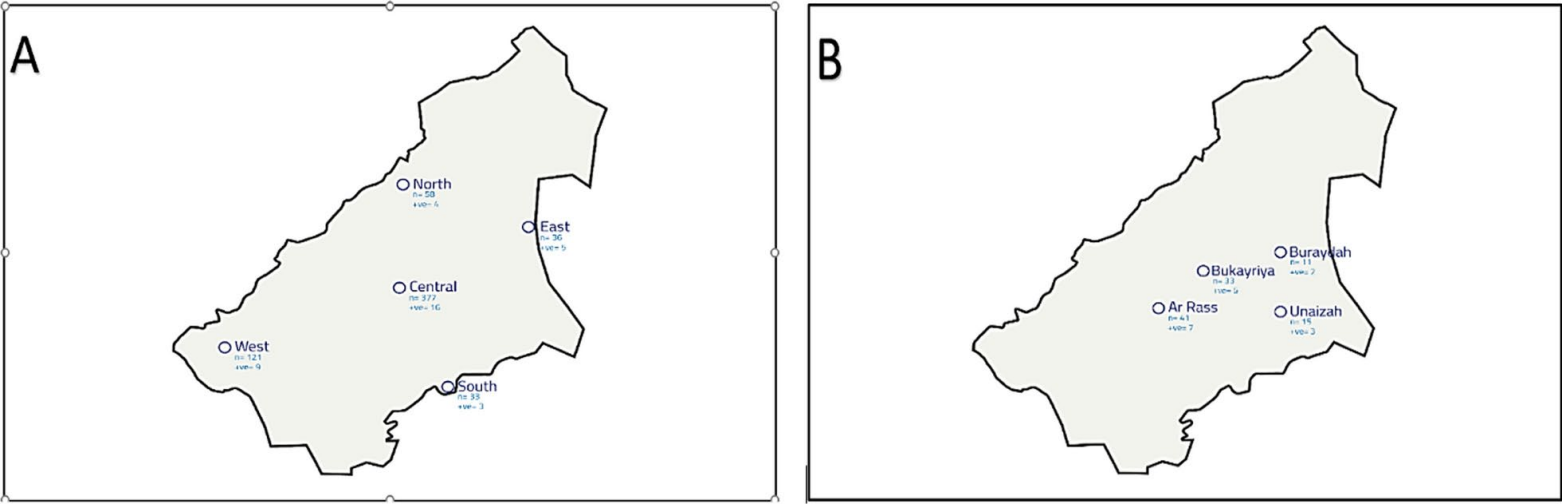
Geographical distributions of brucellosis in camel **A** and human **B** in relation to localities and provinces of the Qassim region

The seroprevalence of bovine brucellosis in camels and humans was assessed through a cross-sectional study design conducted at selected study sites. The sampling methodology was adapted from previously published research (Alatabi et al. 2020; Hassan-Kadle et al. 2021) with modifications. In summary, a total of 625 serum samples from camels were collected in sterile vacuum tubes from specific camel farms and individual animals across various localities in the Al-Qassim region (Table 1 and Fig. 1A). Additionally, 100 serum samples were collected from individuals across various localities and provinces (Fig. 1B). These samples were taken from febrile patients and those referred for brucellosis serodiagnosis at a referral hospital, with collection overseen by a specialist (Table 2).

**Table 1:** Number of camel samples in relation to variables

Variable	No. of samples
*Sex*	
Female	564
Male	61
Total	**625**
*Age*	
≥ 5 year	18
< 5 year	607
Total	625
*Breed*	
Magaheem	219
Homr	33
Sofor	88
Wodh	285
Total	625
*Localities*	
Central (Buraydah)	377
East (Unaizah)	36
West (Ar Rass)	121
South (Almithnab)	33
North (Alasiah)	58
Total	625

**Table 2:** Number of human samples in relation to variables

Variable	No.of samples
*Sex*	
Female	10
Male	90
Total	100
*Age*	
≥ 18 year	72
< 18 year	28
Total	100
*Occupation and infection history*	
Veterinarians	7
Butcher and slaughterhouse workers	8
Camel herders	23
Raw milk consumers	62
Total	100
Buraydah	11
Unaizah	15
Ar Rass	41
Almithnab	19
Alasiah	14
Total	100

### Sample preparation and collection

The collection and preparation of serum from camel and human blood samples were conducted following the methodology outlined by (Alton 1988). In summary, 5 mL of venous blood was collected in sterile vacuum tubes (vacutainers) and subsequently centrifuged at 3000 rpm for 4 min to obtain clear serum. Following this process, 2 mL of the clear serum samples were transferred to Eppendorf tubes and appropriately labeled with the subject’s ID, age, sex, and location. The labeled serum samples were then stored at − 20 °C before serological analysis.

### Sampling technique

Both purposive and randomized sampling techniques were employed for the selection of study animals (camels) and study areas. Given that no prior research has been conducted on brucellosis in camels within the selected regions, samples were randomly collected from farms and veterinary hospitals. Positive cases were isolated and subsequently removed from the population. A total of ten camels that tested positive for brucellosis were slaughtered under official supervision, and tissue specimens—including ten supermammary lymph nodes and ten uterine tissues along with their surrounding fat—were collected immediately postslaughter. These samples were placed in sterile plastic bags and transported to the laboratory on ice for DNA extraction and PCR analysis to detect *Brucella* spp. and perform typing (Singh et al. 2014).

### Antigens for serological tests

The serum samples were analyzed via the RBT and the ARBT as screening tests, in accordance with the guidelines outlined in the OIE Manual (Orr et al. 2022). Both the screening and confirmatory tests, as well as molecular identification, were conducted at the Microbiology Laboratory of the College of Public Health and Health Informatics at Qassim University. Specifically, antibodies against *Brucella* species were detected via the RBT and ARBT, employing commercially available test kits in accordance with the manufacturer's instructions. Sera that tested positive in the RBT were subsequently retested via the ABBEXA *Brucella*—Ab I-ELISA Kit (UK) and the CFT, following the procedures specified by the manufacturer.

### Rose bengal test (RBT)

This test was conducted following the methodology outlined by (Ruiz-Mesa et al. 2005), with certain modifications. In summary, an 8% suspension of *B. abortus* strain 99 was stained with Rose Bengal in a lactate buffer solution (pH = 3.65 ± 0.05), which was procured from commercial kits (Linear Chemicals, Barcelona, Spain).

### Anigen RAPID Brucella Ab Test Kit (ARBT)

The Anigen Rapid Camel *Brucella* Ab Test is a chromatographic immunoassay used for the qualitative detection of *B. melitensis*, *B. abortus,* or *B. suis* antibodies in camel milk, serum, plasma, or whole blood. Camel *Brucella* Ab test strips, disposable capillary tubes, test tubes, anticoagulant tubes, and assay diluents bottles.

### Indirect enzyme-linked immunosorbent assay for *Brucella* (I-ELISA)

*A Brucella* I-ELISA kit was procured from ABBEXA, United Kingdom, for the purpose of testing serum samples from camels. Additionally, a *Brucella* I-ELISA kit was obtained from Cal BIOTECH, United States, to evaluate serum samples from humans. Prior to conducting the tests, the reagents were prepared in accordance with the manufacturer's instructions.

### Complement fixation test (CFT)

All sera that tested positive via the RBT were subsequently subjected to additional testing via a more specific confirmatory CFT with standard *B. abortus* antigen, following the procedures recommended by the World Organization for Animal Health (OIE) to detect the presence of anti-*Brucella* antibodies.

### Molecular characterization and typing of *Brucella* spp.

DNA extraction was performed in accordance with the instructions provided in the gSync™ DNA extraction kit manual (Geneaid, New Taipei City, Taiwan). Conventional PCR was conducted following the methodology outlined by (Bricker 2002) to verify the presence of genetic material belonging to the genus *Brucella*. The amplification of the target gene, specifically the immunodominant antigen gene bp26, was executed in a final reaction volume of 25 µL, which included 12.5 µL of Biomatik® master mix, 1 µL of forward primer, 1 µL of reverse primer (sourced from Biosearch Technologies, South McDowell Boulevard, Petaluma, USA, as referenced by (García-Yoldi et al. 2006)), 7.5 µL of nuclease-free water, and 3 µL of DNA template.

The amplification process was carried out via a Labnet® Multigen Gradient thermal cycler (Labnet International, Inc., Edison, NJ, USA) under the following thermal cycling conditions: initial denaturation at 95 °C for 4 min, followed by 35 cycles of 45 s at 94 °C, 45 s at 60 °C, and 60 s at 72 °C, with a final extension at 72 °C for 7 min. To identify the *Brucella* strain infecting camels, the INgene Bruce ladder (Ingenasa, Madrid, Spain) was utilized, as described by (García-Yoldi et al. 2006). This method employs five primer pairs designed on the basis of strain-specific genetic variations and was implemented in multiplex PCR for molecular typing at the species level. The PCR amplification was again conducted via the Labnet® Multigen Gradient thermal cycler under the same cycling conditions previously mentioned. The PCR amplicons were analyzed by loading 10 µL of the PCR products onto a 1% agarose gel stained with ethidium bromide (0.5 µg/mL). The gels were subsequently photographed under ultraviolet illumination via a gel documentation and analysis system. The identification of *Brucella* species was determined on the basis of the molecular size of the amplified products via a DNA ladder (Biomatik Corporation, Ontario, Canada). The PCR products were visualized through a 1.5% agarose gel stained with ethidium bromide solution (0.5 µg/mL) and documented under an ultraviolet transilluminator.

### Statistical analysis

To achieve the aim of the study, SPSS version 25.0 was used to calculate frequencies, percentages, and chi-square tests under the null hypothesis.

## Results

### Prevalence of brucellosis in the examined camels and humans

The results of the present study indicated that the overall prevalence of brucellosis in camel sera was 9.12% (57 out of 625 samples), as determined by the RBT, and 8.16% (51 out of 625 samples), as determined by the ARBT. Among the camel sera that tested positive for *Brucella* antibodies via RBT, 33 out of 57 samples (57.89%) were also positive according to the i-ELISA, whereas 24 out of 57 samples (42.10%) were positive according to the CFT. Furthermore, the overall prevalence of brucellosis in human sera from febrile patients was 17% (17 out of 100 samples), as determined by the RBT. Among the human sera that tested positive for *Brucella* antibodies via RBT, 12 out of 17 samples (70.58%) were also positive via i-ELISA, and 8 out of 17 samples (47.05%) were positive via CFT, as presented in Table 3.

**Table 3:** Overall prevalence of brucellosis in examined camels and humans in the Qassim region

Source of sample	No. of examined samples	RBT		ARBT		No. of positive samples by RBT	I-ELISA		CFT	
		+ ve	%	+ ve	%		+ ve	%	+ ve	%
Camel	625	57	9.12	51	8.16	57	33	57.89	24	42.10
Human	100	17	17.00	ND		17	12	70.58	8	47.05

*ND* not done

### Prevalence of brucellosis in camels and humans in relation to sex

The seroprevalence of brucellosis in camel sera according to sex was 9.12% (52 positive cases out of 564) in females and 8.19% (5 positive cases out of 61) in males, as determined by RBT. In addition, 8.15% (46 positive cases out of 564) in females and 8.19% (5 positive cases out of 61) in males, as determined by ARBT. Moreover, the data revealed that 53.84% (28 out of 52 positive cases) of the samples were from females, 100% (5 out of 5 positive cases) were from males, as determined by I-ELISA, and 60% (3 out of 5 positive cases) were determined by CFT (Table 4). Although a higher prevalence was recorded in females than in males, the difference was not statistically significant (*p* = 0.449).

**Table 4:** Seroprevalence of brucellosis in camel and human in relation to sex

Source of sample	Sex	No. of examined samples	RBT		ARBT		No. of positive samples by RBT	i-ELISA		CFT	
			+ ve	%	+ ve	%		+ ve	%	+ ve	%
Camel	Female	564	52	9.21	46	8.15	52	28	53.84	21	40.38
	Male	61	5	8.19	5	8.19	5	5	100	3	60.00
	Total	625	57	9.12	51	8.16	57	33	57.89	24	42.10
Human	Female	10	1	10.00	ND		1	1	100	1	100
	Male	90	16	17.77			16	11	68.75	7	43.75
	Total	100	17	17.00			17	12	70.58	8	47.05

*ND* not done

The seroprevalence of brucellosis in human sera concerning sex revealed that 10% (1 positive case out of 10) was recorded in females and that 17.77% (16 positive cases out of 90) was reported in males as determined by RBT. Moreover, the data revealed that 100% of the cases (1 out of 1 positive case tested by RBT) and 87.5% (11 out of 16 positive cases tested by RBT) were recorded in females and males, respectively, as determined by I-ELISA. In addition, 100% (1 out of 1 positive case) and 43.75% (7 out of 16 positive cases were tested by RBT) of the patients were seropositive for brucellosis in females and males, respectively, as determined by the CFT (Table 4). These data revealed that a greater prevalence was recorded in human males than in females, but there was no statistically significant sex difference (*p* = 0.510).

### Prevalence of brucellosis in camels and humans with respect to age

The seroprevalence of brucellosis in camel sera, stratified by age, indicated that 8.73% (53 out of 607 positive cases) and 7.74% (47 out of 607 positive cases) of seropositive individuals were recorded in the age group of 5 years and older. In contrast, *Brucella* antibodies were detected in individuals aged less than 5 years at a rate of 22.22% (4 positive cases out of 18), as determined by both the RBT and the ARBT. Furthermore, the data demonstrated that 56.06% (30 out of 53 positive cases identified by RBT) of seropositive cases were found in the age group of 5 years and older, as determined by i-ELISA, whereas 39.62% (21 out of 53 positive cases identified by RBT) were determined by the CFT (Table 5).

**Table 5:** Seroprevalence of brucellosis in camel and human in relation to age

Source of sample	Age	No. of examined samples	RBT		ARBT		No. of + ve samples by RBT	c- ELISA		CFT	
			+ ve	%	+ ve	%		+ ve	%	+ ve	%
Camel	≥ 5 years	607	53	8.73	47	7.74	53	30	56.06	21	39.62
	< 5 years	18	4	22.22	4	22.22	4	3	75.00	3	75.00
	Total	625	57	9.12	51	8.16	57	33	57.89	24	42.10
Human	≥ 18 years	72	11	15.27	ND		11	9	81.81	6	54.54
	< 18 years	28	6	21.42			6	3	50.00	2	33.33
	Total	100	17	17			17	12	70.58	8	47.05

*ND* not done

The seroprevalence of brucellosis in human sera, stratified by age, indicated that 15.27% (11 out of 17 positive cases) were observed in the age group of 18 years and older, whereas 21.42% (6 out of 28 positive cases) were identified in the age group of less than 18 years, as determined by the RBT. Furthermore, the data revealed that 81.81% (9 out of 11 positive cases identified by RBT) of the patients were aged 18 years and older, whereas 50.00% (3 out of 6 positive cases identified by RBT) of the patients aged less than 18 years were positive, as assessed by i-ELISA. Additionally, 54.54% (6 out of 11 positive cases identified by RBT) were detected in the age group of 18 years and older, whereas 33.33% (2 out of 6 positive cases identified by RBT) were found in the age group of less than 18 years, as determined by the complement fixation test (CFT) (Table 5).

### Prevalence of brucellosis in camels and humans in localities and provinces within the Qassim Region

The seroprevalence of brucellosis in camel sera across various localities was assessed, revealing the following results: 6.89% (26 positive cases out of 377) in Buraydah; 19.44% (7 positive cases out of 36) in Unaizah; 9.09% (11 positive cases out of 121) in Ar Rass; 15.15% (6 positive cases out of 33) in Al Mithnab; and 13.79% (8 positive cases out of 58) in Al Asiooh, all located within the Qassim region. These findings were determined via the rose bengal test (RBT). Additionally, the seroprevalence rates obtained through the ARBT were 6.63% (25 positive cases out of 377) in Buraydah, 13.88% (5 positive cases out of 36) in Unaizah, 8.26% (10 positive cases out of 121) in Ar Rass, 12.12% (4 positive cases out of 33) in Al Mithnab, and 12.06% (8 positive cases out of 58) in Al Asiooh. Furthermore, the data revealed seropositivity rates of 57.96% (15 out of 26 positive cases) in Buraydah, 57.14% (4 out of 7 positive cases) in Unaizah, 72.72% (8 out of 11 positive cases) in Ar Rass, 50.00% (3 out of 6 positive cases) in Al Mithnab, and 37.5% (3 out of 8 positive cases) in Al Asiooh. The CFT results revealed seropositivity rates of 46.15% (12 out of 26 positive cases) in Buraydah, 42.85% (3 out of 7 positive cases) in Unaizah, 45.45% (5 out of 11 positive cases) in Ar Rass, 33.33% (2 out of 6 positive cases) in Al Mithnab, and 25.00% (2 out of 8 positive cases) in Al Asiooh (Table 6). Overall, the highest prevalence of brucellosis was observed in Unaizah, followed by Al Mithnab, Al Asiooh, Ar Rass, and Buraydah. However, no statistically significant differences in seroprevalence were found among the localities within the Qassim region (*p* = 0.184).

**Table 6:** Seroprevalence of brucellosis in camel and human in relation to localities and provinces of Qassim region

Source of sample	Localities and provinces of Qassim	No. of examined samples	RBT		ARBT		No. of positive samples by RBT	i- ELISA		CFT	
			+ ve	%	+ ve	%		+ ve	%	+ ve	%
Camels	Buraydah	377	26	6.89	25	6.63	26	15	57.96	12	46.15
	Unaizah	36	7	19.44	5	13.88	7	4	57.14	3	42.85
	Ar Rass	121	11	9.09	10	8.26	11	8	72.72	5	45.45
	Al mithnab	33	6	15.15	4	12.12	6	3	50.00	2	33.33
	Al asiooh	58	8	13.79	7	12.06	8	3	37.5	2	25.00
	Total	625	57	9.12	51	8.16	57	33	57.89	24	42.10
Humans	Buraydah	14	3	21.42	ND		3	2	66.66	1	33.33
	Unaizah	15	4	26.66			4	3	75.00	2	50.00
	Ar Rass	41	7	17.07			7	5	71.42	4	57.14
	Al mithnab	19	2	10.52			2	1	50.00	1	50.00
	Al asiooh	14	1	7.14			1	1	100	–	–
	Total	100	17	17			17	12	88.2	8	47.05

*ND* not done

The seroprevalence of brucellosis in human sera across various provinces was assessed, revealing the following results: in Buraydah, a seroprevalence of 21.42% (3 positive cases out of 14) was observed; in Unaizah, the seroprevalence was 26.66% (4 positive cases out of 15); in Ar Rass, the seroprevalence was 17.07% (7 positive cases out of 41); in Al Mithnab, the seroprevalence was 10.52% (2 positive cases out of 19); and in Al Asiooh, the seroprevalence was 7.14% (1 positive case out of 14). These findings were determined via the RBT. Furthermore, the data indicated that the seroprevalence rates determined by i-ELISA were 66.66% (2 out of 3 positive cases) in Buraydah, 75.00% (3 out of 4 positive cases) in Unaizah, 71.42% (5 out of 7 positive cases) in Ar Rass, 50.00% (1 out of 2 positive cases) in Al Mithnab, and 100% (1 out of 1 positive case) in Al Asiooh. Additionally, the CFT revealed seroprevalence rates of 33.33% (1 out of 3 positive cases) in Buraydah, 50.00% (2 out of 4 positive cases) in Unaizah, 57.14% (4 out of 7 positive cases) in Ar Rass, and 50.00% (1 out of 2 positive cases) in Al Mithnab. The data suggest that the highest prevalence of brucellosis was recorded in Unaizah, followed by Buraydah, Ar Rass, Al Mithnab, and Al Asiooh. However, no statistically significant differences in seroprevalence were found among the localities within the Qassim region (*p* = 0.184).

### Prevalence of brucellosis in camels with respect to breed variation

The seroprevalence of brucellosis in camel sera, analyzed concerning breed, indicated the following results: 14.61% (32 positive cases out of 219) in Magaheem, 6.06% (2 positive cases out of 33) in Homr, 7.95% (7 positive cases out of 88) in Sofor, and 5.64% (16 positive cases out of 285) in Wodh, as determined by the RBT. Additionally, the seroprevalence rates determined by the ARBT were 13.24% (29 positive cases out of 219) in Magaheem, 6.06% (2 positive cases out of 33) in Homr, 5.68% (5 positive cases out of 88) in Sofor, and 5.26% (15 positive cases out of 285) in Wodh. Furthermore, the data revealed that 75.00% (24 out of 32 positive cases), 100% (2 out of 2 positive cases), 42.85% (3 out of 7 positive cases), and 25.00% (4 out of 16 positive cases) of seropositive cases identified by RBT were recorded in Magaheem, Homr, Sofor, and Wodh, respectively, as determined by i-ELISA. The CFT further indicated that 46.87% (15 out of 32 positive cases), 50.00% (1 out of 2 positive cases), 28.57% (2 out of 7 positive cases), and 31.25% (5 out of 16 positive cases) of seropositive cases identified by RBT were recorded in Magaheem, Homr, Sofor, and Wodh, respectively. These findings are summarized in the supplementary file (Table S1) and illustrated in Fig. 3. The results demonstrated that the highest prevalence of brucellosis was observed in Homr, followed by Sofor, Magaheem, and Wodh; however, no statistically significant differences among the breeds were found (*p* = 0.250).

### Prevalence of brucellosis in humans with respect to occupation and infection history

The seroprevalence rates of brucellosis in human sera concerning occupation and infection history were 16.66% (2 positive cases out of 12), 12.5% (1 positive case out of 8), 13.04% (3 positive cases out of 23), and 19.29% (11 positive cases out of 57) among veterinarians, butchers and slaughterhouse workers, camel herders, and raw milk consumers, respectively, as determined by the RBT. Furthermore, the data revealed that 100% (2 out of 2 positive cases), 100% (1 out of 1 positive case), 33.33% (1 out of 3 positive cases), and 72.72% (8 out of 11 positive cases) were identified in veterinarians, butchers and slaughterhouse workers, camel herders, and raw milk consumers, respectively, as determined by i-ELISA. Additionally, 100% (2 out of 2 positive cases), 0% (0 out of 1 positive case), 0% (0 out of 2 positive cases), and 54.54% (6 out of 11 positive cases) of seropositive individuals were recorded in veterinarians, butchers and slaughterhouse workers, camel herders, and raw milk consumers, respectively, as determined by the CFT (see supplementary file, Table S2). These findings indicate that the highest prevalence of brucellosis was observed among veterinarians, followed by raw milk consumers, butchers, slaughterhouse workers, and camel herders; however, there was no statistically significant difference among these groups (p = 0.258).

### Molecular identification and typing of *Brucella* spp.

**Fig. 2:**
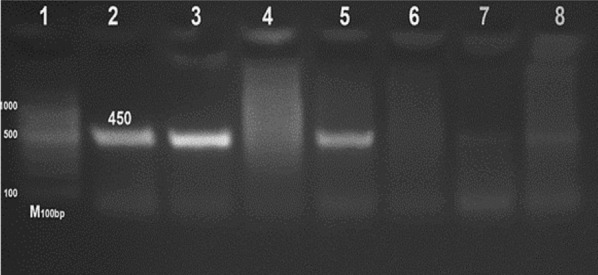
PCR amplification products generated by *Brucella* genus-specific primers

**Fig. 3:**
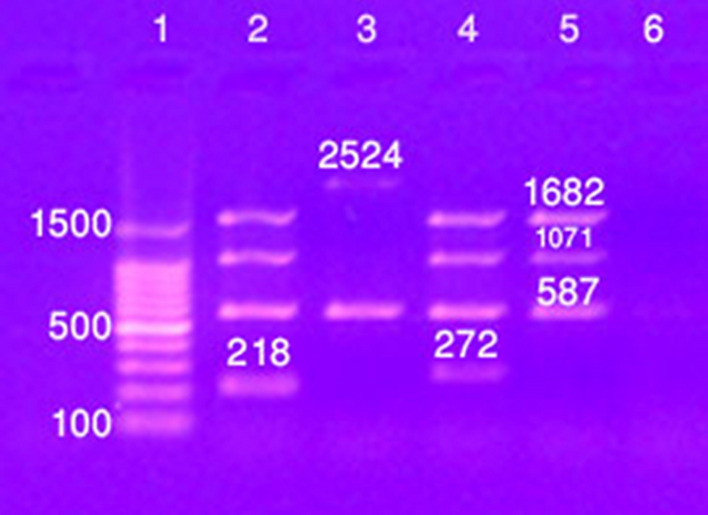
Results of Bruce-ladder PCR analysis on the DNA extract from a *Brucella* isolate. Lane 1: 100 bp DNA ladder; Lane 2: Rev1 (control from the Bruce-ladder kit); Lane 3: RB51 (control from the Bruce-ladder kit); Lane 4: Suis (control from the Bruce-ladder kit); Lane 5: *Brucella* isolate; Lane 6: negative control

A clear band at 450 base pairs was detected as a PCR product from three samples of ten serologically positive confirmed cases, indicating the presence of *Brucella* spp. (Fig. 2). The *Brucella* strain was identified on the basis of the molecular size of the amplified products via a DNA ladder, which indicated that the *Brucella* isolate recovered in this study was *B. melitensis* biovar 3 (Fig. 3).

## Discussion

Camels play a significant role in the transmission of brucellosis to humans, which results in serious health and productivity issues. Brucellosis is common in camels and humans in Qassim, Saudi Arabia, where it poses public health risks from raw milk consumption and contact with aborted animals. To protect public health, it is vital to manage risk factors, provide *Brucella* diagnostics, offer ongoing training, and adopt the One Health approach to increase health for both people and domestic animals. Brucellosis is caused primarily by various species of the *Brucella* genus, exhibiting widely varying prevalence rates across different countries, both in animal and human populations (Nguna et al. 2019; Almuzaini 2023). The genus *Brucella* is classified as a gram-negative coccobacilli, which poses a considerable threat to public health (Ran et al. 2019; Selim et al. 2019). Notably, brucellosis remains the most prevalent zoonosis in the Eastern Mediterranean region, particularly in developing countries such as Saudi Arabia (Al-Eissa 1999; Dean et al. 2012). The seroprevalence of Brucella antibodies can be exceptionally high, especially among populations residing in endemic areas. During the 1990s, the Kingdom of Saudi Arabia (KSA) reported the highest prevalence of human brucellosis in the Middle East (Ali 2009). Brucellosis constitutes an occupational hazard for individuals at heightened risk, including laboratory personnel, veterinarians, abattoir workers, farmers, and animal caretakers (Shoukat et al. 2017).

The overall seroprevalence of brucellosis in camels observed in this study was higher than that reported by (Megersa et al. 2012) and (Gizaw et al. 2017) but lower than that reported by (El-Boshy et al. 2009; Zewolda and Wereta 2012; Alatabi et al. 2020). Variations in prevalence or incidence rates among different animal species may be attributed to several factors, including sample size, production systems, breed differences, age, sex, climate, environmental conditions, and geographical locations (Terefe et al. 2017; Khan et al. 2020). Furthermore, factors such as animal risk profiles, farm management practices, geographical distribution, herd management, and farmer awareness of brucellosis have been linked to the prevalence of the disease (Coelho et al. 2015). Additionally, the cohabitation of camels with other livestock species has been identified as a significant risk factor for the transmission of brucellosis in camels (Fatima et al. 2016; Bayasgalan et al. 2018).

The present study revealed a notably high seroprevalence of brucellosis among febrile patients. This finding surpasses the rates reported by (Tadesse 2016). The elevated seroprevalence may be attributed to the fact that the human samples examined were specifically selected from individuals experiencing fever, who were referred by their attending physicians for serodiagnosis of brucellosis at a referral hospital. According to (Asaad and Alqahtani 2012) and (Bayasgalan et al. 2018), seroprevalences of brucellosis exceeding 5% in either animals or humans are significant and suggest an endemic status. Furthermore, the Al‒Qassim region is characterized as highly enzootic, which may account for the observed variation (Wareth et al. 2014). Various demographic, occupational, and socioeconomic factors may contribute to the incidence of brucellosis (Pappas et al. 2006; Wareth et al. 2014). Therefore, the implementation of comprehensive and effective public health education campaigns is crucial for the prevention of camel brucellosis and the enhancement of human health management.

The study revealed a higher seroprevalence of brucellosis in female camels than in male camels; however, this difference was not statistically significant (*p* = 0.449). This finding aligns with the results reported by (Patel et al. 2017), who also noted a greater seroprevalence of brucellosis in female camels. Conversely, (Tadesse 2016) reported a tendency for females to exhibit slightly greater seropositivity than males do, attributing this to the longer lifespan of females and their greater population in the sampling procedures. Owing to their utility in production herds, female camels generally have a longer lifespan than males do, potentially leading to increased exposure to the bacterium (Mekonnen et al. 2010). In contrast, other studies, such as that by (Khan et al. 2020), reported a higher seroprevalence in males than in females. This discrepancy in infection rates may be attributed to the predilection of *Brucella* for reproductive organs, as well as the fact that it is young (Acha and Szyfres 2001). Compared with females, male camels may face a greater risk of infection, as they are often exposed to the pathogen both within and outside the herd while seeking mates.

This study revealed a higher seroprevalence of human brucellosis in males than in females; however, this difference was not statistically significant (*p* = 0.510). This finding is consistent with the observations of (Ageely et al. 2016; Alsoghair 2017), who reported a significantly higher prevalence of human brucellosis in males than in females. Furthermore, (Kairu-Wanyoike et al. 2019) noted that the seroprevalence of *Brucella* was significantly elevated among males, older individuals, and those residing in pastoral areas. Conversely, (Malik 1997) and (Dawood et al. 2023) reported a higher seroprevalence of brucellosis in females than in males; nonetheless, this difference was also not statistically significant (*p* = 0.872), as reported by (Al-Sekait 2000; Mangalgi et al. 2015; Terefe et al. 2017). These discrepancies are likely attributable to variations in occupational exposures, practices, and habits among the different populations studied (Al-Tawfiq and AbuKhamsin 2009).

A high prevalence of seropositive sera for brucellosis was observed in camels aged ≤ 5 years and in humans aged ≤ 18 years. This phenomenon may be attributed to the tendency for brucellosis seropositivity to increase with increasing age. It is important to note potential biases in the data, as the majority of the tested human sera were derived from male subjects, whereas older female animals were predominantly represented. These findings are consistent with those reported in the literature (Fatima et al. 2016; Terefe et al. 2017; Bayasgalan et al. 2018). Furthermore, a meta-analysis conducted by (Tadesse 2016) indicated that the calculated probability value suggested that postpubertal or older animals are more likely to be seropositive for brucellosis than younger or prepubertal animals are, again highlighting the influence of age. Additionally, a study by (Alkahtani et al. 2020) revealed that individuals aged 21–40 years presented a relatively high prevalence of brucellosis, whereas older adults and young children presented the lowest prevalence rates. The observed increase in the seroprevalence of brucellosis with age aligns with findings from studies conducted in Iran, Jordan, Lebanon, and Kuwait (Lulu et al. 1988; Araj and Azzam 1996). However, it is noteworthy that with advancing age, the risk factors associated with occupation and infection history also tend to increase.

The study indicated that the highest seroprevalence of brucellosis in camels was observed in Unaizah, followed by Al Mithanb, Al Asioon, Ar Rass, and Buraydah; however, no statistically significant differences among these localities were found (*p* = 0.184). This lack of significance may be attributed to the larger population of camels in Unaizah than in the other localities. Conversely, Bayasgalan et al. reported that seropositivity was significantly greater in camels from eastern Mongolia than in those from western and southern provinces, which was attributed to the cohabitation of cattle and camels (Bayasgalan et al. 2018). Furthermore, camel herds that employed an open replacement system and had a history of abortions were found to be more likely to test positive for brucellosis (Alrawahi et al. 2019). A larger herd size increases the likelihood of contact among infected animals, particularly in instances where abortions have occurred (Abbas and Agab 2002). Additionally, camels that are kept in proximity to small ruminants are also more susceptible to infection, as evidenced by various studies (Ismaily et al. 1988; Radwan et al. 1992; Abou-Eisha 2000; Al-Majali et al. 2008).

The data indicated that the highest seroprevalence of human brucellosis was observed in Unaizah, followed by Buraydah, Ar Rass, Al Mithanib, and Al Asioon. However, there was no statistically significant difference in seroprevalence among the provinces in the Qassim region (*p* = 0.980). This lack of significant difference may be attributed to the greater number of camel herds in Unaizah than in the other provinces. Conversely, Alsoghair reported that the highest incidence of human brucellosis occurred in Uyoon Al-Jawa, followed by Buraydah within the Qassim region (Alsoghair 2017). The variations in brucellosis prevalence rates across different geographical regions may be associated with disparities in disease prevalence among animal populations, occupational exposure, or differing social behaviors within various populations (Lulu et al. 1988; Araj and Azzam 1996).

The highest seroprevalence of camel brucellosis was observed in the Magaheem breed, followed by the Sofr, Homr, and Wodh breeds; however, the differences among the breeds were not statistically significant (*p* = 0.250). Additionally, Alrawahi and colleagues reported that all seropositive cases of camel brucellosis were attributed to local breeds (Alrawahi et al. 2019). The Magaheem breed is recognized as the most prevalent breed in Saudi Arabia and had the largest sample size examined among the various breeds. Factors such as the composition of livestock species, frequency, and range of animal mobility, as well as herd or flock size, may contribute to the observed differences in the seroprevalence of brucellosis (Tadesse 2016). Furthermore, the disparities in brucellosis prevalence among breeds, along with management and husbandry practices, are likely more critical than breeding alone in determining the likelihood of a camel being infected with *Brucella* (Radwan et al. 1995).

The present study revealed the highest prevalence of human brucellosis among consumers of raw milk, followed by veterinarians, camel herders, and workers in butcheries and slaughterhouses, with no statistically significant difference observed (*p* = 0.258). This finding aligns with the observations of Migisha and colleagues, who indicated that the consumption of raw milk is a primary factor independently associated with brucellosis (Migisha et al. 2018). Reports of human brucellosis outbreaks linked to the consumption of infected raw camel milk have been documented in various countries (Garcell et al. 2016). Although the biases present in the sample distribution may influence the results, the data nonetheless suggest a heightened transmission rate of *Brucella* spp. to humans from animals through consumption and other forms of contact (Tadesse 2016). Furthermore, a history of direct contact with infected animals and the consumption of raw milk are recognized as predisposing factors associated with the prevalence of human brucellosis (Minas et al. 2007; Alhoshani et al. 2016; Fatima et al. 2016; Alsoghair 2017). Additionally, the frequent consumption of raw milk, as well as the roles of shepherds and individuals over the age of 30 who have daily contact with animals, significantly increase the risk of brucellosis (Al-Hakami et al. 2019). Infection with *Brucella* in humans poses an occupational risk for abattoir workers, farmers, laboratory personnel, veterinarians, and individuals who work with animals or consume animal products (Smits et al., 2004). In Saudi Arabia, the consumption of camel milk is a common practice and tradition; however, no correlation has been established between this practice and brucellosis seropositivity (Ageely et al. 2016).

Owing to the high sensitivity and increased incidence of false positive reactions in large ruminants, the current guidelines established by the World Organization for Animal Health (OIE) recommend that all positive results from the RBT be confirmed through quantitative assays, including the CFT and ELISA (Corbel and Moriyon 2006). Consequently, in this study, the i-ELISA and CFT methodologies were employed to further evaluate the serological results obtained from the RBT. The diagnosis of brucellosis necessitates laboratory screening and confirmation via widely accepted bacteriological, molecular, and serological techniques that exhibit high sensitivity and specificity, which are essential for any control or eradication program. Bacteriological and immunological tests serve as the primary diagnostic tools for brucellosis in camels (Wareth et al. 2021). The discrepancies observed between these two testing methods may be attributed to various factors, including differences in sensitivity and specificity; however, it has been established that i-ELISA has greater sensitivity than RBT does, whereas RBT has greater specificity than i-ELISA does (Madzingira and Sezuni 2017; ElTahir et al. 2019). Nevertheless, RBT results must be confirmed through CFT or ELISA, as recommended by the OIE.

The classical RBT and the ARBT serve as initial screening tests, whereas additional assays such as the SAT, CFT, and ELISA are employed as confirmatory serological tests. In serological diagnostics, a screening test characterized by high sensitivity is typically succeeded by a confirmatory test with high specificity. Various methods, including RBT, CFT, SAT, competitive ELISA (c-ELISA), fluorescence polarization assay (FPA), and i-ELISA, have been utilized for the detection of anti-*Brucella* antibodies in camel sera (Fatima et al. 2016). The ELISA test is also capable of providing an approximate assessment of the disease stage and distinguishing between the presence of specific immunoglobulin M and immunoglobulin G antibodies (Terefe et al. 2017). Among the available methods, direct agglutination, the Rose Bengal test, and c-ELISA are considered the most effective for the serological diagnosis of brucellosis (Sanogo et al. 2013).

The RBT is widely recognized as the most prevalent diagnostic method for detecting camel brucellosis, primarily because of its reliable results (Bayasgalan et al. 2018). However, a significant limitation of RBT is its propensity to yield a high number of false positive results, which are often attributed to cross-reactivity with other bacterial species (Zakaria et al. 2018). Among the four diagnostic tests employed for camel brucellosis, the immunochromatographic test (ICT) demonstrated the highest sensitivity, followed by FPA, c-ELISA, and finally the RBT. Conversely, there were no statistically significant differences in specificity among the four tests, with the ICT exhibiting the lowest specificity and the RBT showing the highest specificity (Serhan et al. 2019). Notably, while the c-ELISA test is characterized by lower sensitivity, it is the most specific method for confirming the presence of Brucella antibodies (Madut et al. 2018) and serves as an appropriate confirmatory test following the RBT (Alatabi et al. 2020).

The PCR assay has been demonstrated to be an effective method for the detection of Brucella organisms in various tissues, as reported by (Ouahrani‐Bettache et al. 1996). Numerous advantages of the PCR assay over conventional methods for identifying *Brucella* species have been documented. A primary advantage is the significantly reduced time required for detection, as conventional methods typically require several days for the isolation and identification of the organism (Fekete et al. 1992; Bricker and Halling 1994; Ouahrani‐Bettache et al., 1996; Bricker et al. 2000). Additionally, the PCR assay requires minimal sample preparation, as it does not necessitate the isolation of viable organisms. Furthermore, the assay is not adversely affected by contamination from other microbial species that may be present in the tissue samples utilized for isolation.

In conclusion, camels are a reservoir for brucellosis in humans and are transmitted through raw milk and direct contact. The highest risk is for veterinarians, camel herders, butchers, and slaughterhouse workers. Female camels in the central Qassim region, particularly the Magaheem breed, presented the highest seroprevalence during the winter. Human brucellosis is most prevalent among males in the winter and among raw milk consumers, likely because of their greater consumption of raw milk. To reduce brucellosis incidence in humans and camels, interventions should include public awareness campaigns, protective clothing for handling aborted she-camels, and milk pasteurization. Additional measures include livestock vaccination, hygiene practices, quarantine protocols, and serological testing to identify infected animals. Strengthening collaboration between health authorities and the veterinary sector is vital. Further research is needed to distinguish between vaccinated and nonvaccinated camels, and standardizing serological tests for diagnosing brucellosis is essential.

## Limitations of the study

Some owners of camel farms declined to permit the collection of blood samples from their herds, indicating that such practices would negatively impact animal productivity. As a result, the target sample size was not achieved. Furthermore, owing to the restricted number of districts included in the study, the findings cannot be generalized to all regions of Saudi Arabia.

## Supplementary Information

Supplementary Material 1.

## Data Availability

This published article and supplementary file include all data generated, and, or analyzed during this present study.
