# Supplementary material for: Seroprevalence of brucellosis in camels and humans in the Al-Qassim region of Saudi Arabia and its implications for public health
Source: AMB Express. 2025 Feb 7;15:22. doi: 10.1186/s13568-025-01822-8 (PMC11806190; doi:10.1186/s13568-025-01822-8)
Supplement: Supplementary file 1 — Supplementary Material 1. [file 13568_2025_1822_MOESM1_ESM.docx]

**Supplementary file**

**Table S1.** Seroprevalence of brucellosis in camels in relation to breeds

| Breed | No. of examined samples | RBT | | ARBT | | No. of +ve samples by RBT | I- ELISA | | CFT | |
| --- | --- | --- | --- | --- | --- | --- | --- | --- | --- | --- |
|  |  | +ve | % | +ve | % |  | +ve | % | +ve | % |
| Magaheem | 219 | 32 | 14.61 | 29 | 13.24 | 32 | 24 | 75.00 | 15 | 46.87 |
| Homr | 33 | 2 | 6.06 | 2 | 6.06 | 2 | 2 | 100 | 1 | 50.00 |
| Sofor | 88 | 7 | 7.95 | 5 | 5.68 | 7 | 3 | 42.85 | 2 | 28.57 |
| Wodh | 285 | 16 | 5.64 | 15 | 5.26 | 16 | 4 | 25.00 | 5 | 31.25 |
| Total | 625 | 57 | 9.12 | 51 | 8.16 | 57 | 33 | 57.89 | 24 | 42.1 |

**Table S2.** Seroprevalence of brucellosis in humans in relation to occupation and infection history

| Occupation and infection history | No. of febrile patients | RBT | | No. of +ve samples by RBT | c- ELISA | | CFT | |
| --- | --- | --- | --- | --- | --- | --- | --- | --- |
|  |  | +ve | % |  | +ve | % | +ve | % |
| Veterinarians | 12 | 2 | 16.66 | 2 | 2 | 100 | 2 | 100 |
| Butcher and Slaughterhouse workers | 8 | 1 | 12.5 | 1 | 1 | 100 | - | - |
| Camel herders | 23 | 3 | 13.04 | 3 | 1 | 33.33 | - | - |
| Raw milk consumers | 57 | 11 | 19.29 | 11 | 8 | 72.72 | 6 | 54.54 |
| Total | 100 | 17 | 17.00 | 17 | 12 | 70.08 | 8 | 47.05 |
